# Analysis of Gene Expression and Neuronal Phenotype in Neuroscreen-1 (NS-1) Cells

**Published:** 2018-12-17

**Authors:** Smritee Pokharel, Chang Hun Lee, Nailya Gilyazova, Gordon C. Ibeanu

**Affiliations:** 1 Biomanufacturing Research Institute and Technology Enterprise (BRITE), North Carolina Central University, Durham, North Carolina, United States of America; 2 School of Dentistry, Medical College of Virginia, Richmond, Virginia, United States of America; 3 Department of Pharmaceutical Science, North Carolina Central University, Durham, North Carolina United States of America

**Keywords:** Neuroscreen-1, Pheochromocytoma, NS-1, PC12, Neuronal, Neurite, Transcription, Cholinergic, Differentiation, Nerve Growth Factor

## Abstract

Neuroscreen-1 (NS-1) a sub-clone of pheochromocytoma (PC12) cell is gaining broad acceptance as in vitro neuronal model for biochemical and phenotypic assays due to robust growth and differentiation profiles. However, the molecular characteristics of the cell remains to be documented. In this study, we performed comparative analysis for expression of neuronal marker genes in undifferentiated and nerve growth factor (NGF) differentiated NS-1 and PC12 by qPCR and immunoblot assays. We show that differentiation of NS-1 occurred under low concentrations of NGF relative to PC12. Cell growth also occurred more rapidly in NS-1. Transcriptional analysis of neuronal marker genes showed comparable expression of tyrosine receptor kinases (Ntrk1, Ntrk2, NGFR/p75NTR) and muscarinic acetylcholine (Chrm1, Chrm2, Chrm3, Chrm4) receptors in unspecialized cells. Ntrk2, adenosine receptors (Adora1, Adora2A) and choline acetyltransferase (ChAT) were altered in undifferentiated NS-1. In contrast, Ntrk1, Ntrk2, Chrm2 transcripts were vastly increased in NS-1 with NGF exposure, while Ntrk3, Adora1 and Adora2A transcripts were reduced. In differentiated PC12, Chrm4 and ChAT were markedly upregulated. Our data suggests that differences in morphological and phenotypic characteristics that distinguish NS-1 from PC12 is likely the product of altered gene expression. Furthermore, expression of neuron type genes in NS-1 support its use as an alternative model to PC12.

## Introduction

Development of *in vitro* disease model that closely mimics *in vivo* condition has become significant with the emergence of new disease and pathological conditions. Basic research for neurodegenerative disease largely relies on cell line studies, which also act as the primary platform for drug screening in translational medicine. However, complex nutritional requirements, slow growth properties, and expression of cell-type specific markers limit the choice of cell lines used in research. Neuroscreen-1 (NS-1), is a sub-clone of PC12, a noradrenergic clonal line of adrenal pheochromocytoma that shows dopaminergic properties and are widely used in neurobiology, neurotoxicology, and drug discovery studies [1–5].

Differentiation of PC12 by NGF is well documented. NGF treatment of PC12 cell leads to cessation of cell division, induction of neurite outgrowth, and production of electrically excitable cells in culture, mimicking characteristics of sympathetic-like neurons [1–6]. In addition to the neuroprotective and neuro-restorative properties of NGF, dysregulation in NGF signaling has been positively correlated with neurodegenerative disease including Alzheimer’s disease (AD) [7–8], epilepsy [9] and cancer [10,12]. AD is characterized by death of forebrain cholinergic neurons that provides cholinergic innervations to cerebral cortex and hippocampus. Neuritic plaques comprising β-amyloid fibrils, dystrophic neuritis, reactive astrocytes, phagocytic cells and protein fragments derived from degenerating neurons are the typical feature of AD. NGF regulates proliferation and differentiation of neuronal cells via activation of activation of tyrosine protein kinase (TrkA) receptor, and downstream signaling molecules that include Ras/MAP kinase cascade, IP3-dependent Ca2+ release, and PI3K/Akt pathways [13]. TrkA also enhance neuronal survival by silencing the tumor necrosis factor receptor family member p75^NTR^ [14]. Furthermore, NGF increases expression of ChAT and VAChT, two cholinergic specific markers that are required for cholinergic neurotransmission [15–16]. ChAT enables the synthesis of acetylcholine (ACh) from acetyl-CoA and choline, whereas VAChT acts as a membrane transporter loading ACh into secretory vesicle and makes it available for secretion [17]. Decrease in ChAT and VAChT is suggested to play a role in the progression of AD [18–19].

Muscarinic acetyl choline receptors (Chrms) are members of the G-Protein coupled receptor family, expressed in both the central nervous system (CNS) and peripheral nervous system (PNS). Chrms comprise of five genes (*Chrm1, Chrm2, Chrm3, Chrm4* and *Chrm5*) which specify five membrane receptor subtypes (M1-M5) divided into two broad classes based on their coupling efficiency with G proteins. Muscarinic receptors found in the cholinergic system signal through the binding of acetylcholine and are thought to be involved in learning, memory [20,21], motor and sensory modulation [22] and thus has been implicated in a wide variety of physiological processes in the CNS and diseases such as Alzheimer’s disease and schizophrenia [23] Parkinson’s disease [24]vascular dementia and depression [25]. Similar to muscarinic receptors, the adenosine receptors (AR) are GPCRs present in the CNS and mediate the actions of adenosine in the release of neurotransmitters and synaptic plasticity [26]. There are four members of the family, A_1_, A_2A_, A_2B_, and A_3_. Adenosine A_1_, A_2A_, and A_2B_ receptor mRNAs have been detected in PC12 variants. Adenosine A_1_ and A_2A_ receptors are present in the microglia and neurons, have relatively high affinity for adenosine, and demonstrate opposing actions in the CNS. A_1_ receptors are prevalent in the synaptic regions whereas A_2A_ receptor is localized in the striatum and olfactory bulb [27]. The A_2A_ receptor has been shown to modulate NGF-induced neurite outgrowth (NOG) in PC12 and neuritogenesis in primary hippocampal neuron in association with translin-associated protein X (TRAX) [28].

NS-1 cell line has been used as a neuronal cell model to study the chemical dependent initiation, progression, inhibition, and toxicity in neurite outgrowth assays [2,5,29,30]. Despite increasing interest in the use of NS-1 as a substitute model for PC12, differences in growth and differentiation characteristic have not been studied at the molecular level. No comparative information exists in the literature on gene expression patterns between NS-1 and the parental PC12. Understanding traits of NS-1 gene expression in differentiated and naïve states is critical in defining the representative neuronal subtype for utility as a relevant substitute experimental model for PC12. In the present study, we characterized the gene and protein expression profiles by comparative analysis of neuronal molecular markers by qPCR and immunoblotanalyses. We conclude that changes in gene and protein expression could account for enhanced phenotypic properties of NS-1, and that the expression of neuronal markers support the use of NS-1 as an alternative and substitute cell model to PC12.

## Materials and Methods

Reagents were sourced from the following vendors: NS-1 (Cellomics, Inc., Pittsburgh, PA), PC12 (ATCC, Manassas, VA), 2.5S murine NGF (Bachem Inc., Torrance, CA), RNA extraction kit and Turbo DNA-free kit and RETROscript Kit (Ambion Inc., Carlsbad, CA), RT PCR primers (Eurofins MWG Operon, Huntsville, AL); RT^2^ SYBR Green ROX qPCR Mastermix (QIAGEN, Valencia, CA), MTT (3-(4, 5-dimethylthiazolyl-2)-2,5-diphenyltetrazolium bromide) (Sigma-Aldrich, St. Louis, MO), DMEM and RPMI 1640, HCS CellMask Red™, and Alexa Fluor 488 Donkey anti-rabbit antibody (Invitrogen, Carlsbad, CA), fetal bovine serum (FBS) (Hyclone, Logan, UT), heat inactivated horse serum (Lonza, Walkersville, MD), and L-glutamine and 1% Penicillin/Streptomycin (BioWhittaker, Walkersville, MD), Mouse anti-ChAT and Rabbit anti-M2 antibody (EMD Millipore, Billerica, MA), Goat anti-VAChT (Promega, Madison, WI), Rabbit anti-TrkA (Cell Signaling, Tech., Danvers, MA), and Super Signal Pico Chemiluminescent Substrate (Thermo Fisher Sci., Grand Island, NY).

### Cell culture and differentiation

PC-12 cells were maintained in collagen coated plates in DMEM containing 5% FBS, 10% heat inactivated horse serum, 1% L-glutamine and 1% Penicillin/Streptomycin. NS-1 cells were maintained in RPMI-1640 supplemented with 10% FBS, 1% L-glutamine and 1% Penicillin/Streptomycin. The culture media was renewed every three days and cells sub-cultured at 80% confluence. Cell differentiation was accomplished in the presence of 20ng/mL NGF in reduced serum media containing 2% FBS. Following differentiation, cells were observed for change in morphology characterized by presence of elongated neurites. For dose response assays, NS-1 and PC-12 cells were treated with NGF in reduced serum media containing 0, 3.13, 6.25, 12.5, 25 and 50 ng/ml of NGF respectively for 48 h followed by staining, capture of image and quantification of the neurite.

### Analysis of neurites

The quantification of neurites was performed as described previously reported [5] with minor changes. Briefly, NS-1 cells were seeded in 384 well plate at a density of 5×10^2^ cells per well and allowed to grow in the presence of 0, 3.13, 6.25, 12.5, 25, and 50 ng/mL NGF respectively for 48 hrs. At neurite maturity, the differentiation media was aspirated from and replaced with 30 μL of staining solution containing 0.2μg/ml Hoechst dye and 0.25μg/ml HCS CellMask Red™ (HCMR) in 4% formaldehyde. The plate was incubated in the dark at 23°C for 1 hour to obtain fixed samples with Hoechst-stained nuclei and HCMR-stained cell bodies and extensions. The staining solution was aspirated, cells rinsed with PBS, and images were acquired on a CellInsight™ CX5 High Content Screening (HCS) Platform. Images were captured by 10X objective in two different channels: Nuclei (Hoechst) with excitations of 386/23 nm, Cell body/ neurites (HCS Red) 650/13 nm. Neurites were analyzed using HCS Studio™ Cell Analysis Software- Neuronal Profiling v3.5 BioApplication (Thermo Fisher Scientific™).

### RNA isolation and RT-qPCR

Total RNA isolated using Ambion RNA extraction kit was reverse transcribed with the RETROscript reverse transcription kit to obtain complementary DNA (cDNA). Real time-qPCR was conducted with 2 μL cDNA, 2 μL of 10 μM gene specific primer, 10 μL (2X) SYBR Green reagent and 6 μL DNase/RNase free water. The amplification protocol entailed 40 cycles of denaturation at 94°C for 30 secs, annealing at 55 to 57°C for 30 sec and extension at 72°C for 30 sec, followed by the final extension for 5 min at 72°C in a 7500 RT- PCR system (Applied Biosystems, Foster City, CA). Confirmation of specificity of PCR products was achieved by analysis of melting curves. Genes were analyzed and fold differences were calculated using RPL-19 as reference gene. The primers were designed by Primer 3 software (http://frodo.wi.mit.edu) using the sequence information listed at the National Centre for Biotechnology Information and sequence verification was done using BLAST. Detailed information on primer sequence is provided as a Table 1.

### Immunoblot analyses

Cells cultured in 6 well plates were washed in ice cold PBS and lysed in RIPA buffer containing protease inhibitor cocktail (ThermoFisher Scientific). Total protein was isolated and quantified by Bradford method [31]. A total of 20 μg protein was reduced, electrophoresed in 10% SDS-polyacrylamide gel and transferred to PVDF. Membranes were blocked, incubated with mouse anti-ChAT, goat anti-VAChT, or rabbit anti-TrkA, washed, incubated with species specific secondary antibody and visualized with Super Signal Pico Chemiluminescent Substrate (Thermo Scientific). Band intensity was measured densitometrically using ImageJ software and results were normalized with β-actin as internal control.

### Immunocytochemistry

Cells allowed to grow and differentiate in glass slides in presence or absence of NGF for 24 h were stained as previously described [32]. Briefly, cells were rinsed with PBS, fixed in 4% formaldehyde, permeabilized in 0.2% Triton X-100. Cells were blocked and subsequently incubated with primary rabbit anti-M2 antibody overnight at 4°C. Cells were further treated with secondary antibody (Alexa Fluor 488 Donkey anti-rabbit; Invitrogen, Eugene, OR, USA) followed by nuclei staining with mounting media containing 4’, 6-diamino 2-phenylindole (DAPI; Sigma-Aldrich). Cell pictures were taken using an Olympus inverted fluorescence microscope equipped with a digital camera (Nikon, Tokyo, Japan).

### Statistical analyses

Statistical analyses were performed in GraphPad PRISM 7 (GraphPad, La Jolla, CA, USA). Data are provided as means ± standard error (SEM) of at least three independent experiments. Significant differences between groups were determined using either multiple t-test or two-way ANOVA. A nominal *p*-value of less than 0.05 was considered to be statistically significant

## Results

### Analyses of NGF-dependent neurite outgrowth in differentiated cells

**Figure 1:**
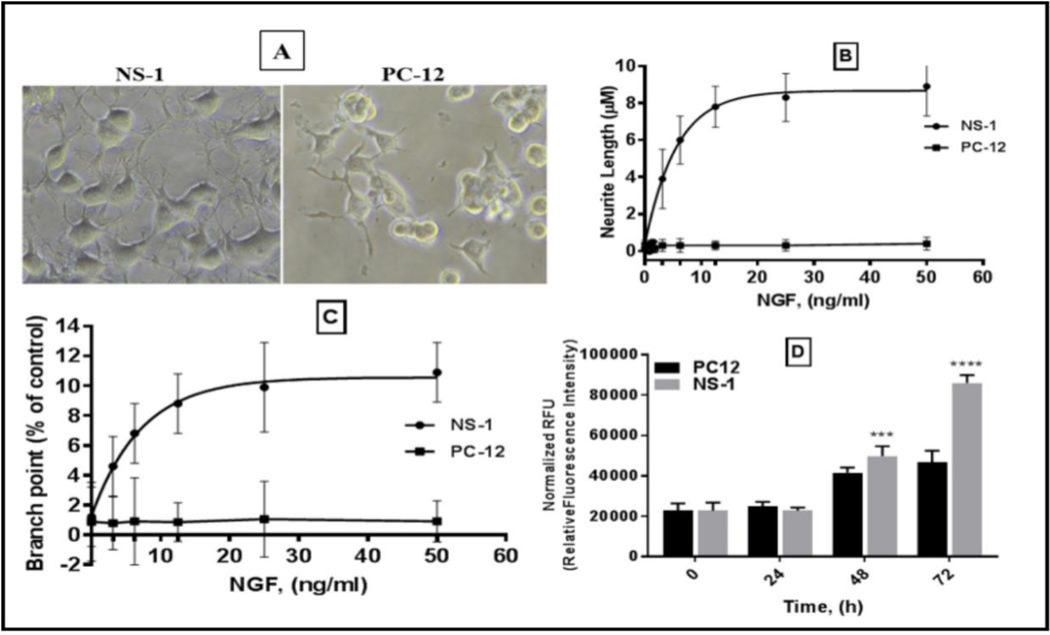
Morphometric analysis of NGF induced neurite outgrowth in NS-1 and PC12. Cells were treated with increasing concentrations of NGF for 48 h processes with HCS Red™ and Propidium Iodide and analyzed for neurite length and branch points on the Cellomics imaging platform. (A) Phase contrast photomicrographs of NS-1 cells (top left panel) and PC12 (top right panel) treated with 50 ng/mL NGF showing presence of neurites and branch points. (B) Graphical quantification of NGF concentration-dependent stimulation of neurite lengths (top) and branch points (bottom) in NS-1 and PC12. (C) Graph of growth analysis of NS-1 and PC12 cells acquired at various time points after inoculation in media. (***p<0.001; ****p<0.0001 indicates significance).

NS-1 is a clonal line of the pheochromocytoma cell PC12, which has the ability to differentiate into neuronal-like cells in presence of NGF. NS-1 and PC12 cells exhibit phenotypic and functional characteristics associated with NGF differentiation in altered timescales. To explore the underlying causes for these differences in characteristics we began by evaluating the NGF-dependent neurite growth dynamics in NS-1 and PC12 cells seeded overnight in growth media and treated with increasing concentrations of NGF. Morphological appearance of the cells was assessed visually and quantified for neurite length and branch points after 48 h in culture. NGF treatment induced hypertrophy in NS-1 and PC12 and produced cells with enhanced neuron-like morphological characteristics. NGF stimulation led to increase in cell volume, change in shape and appearance, and formation of neuritic processes (Figure 1A, upper panel).

Differentiated NS-1 developed dense neurite networks characterized by long neurites seen in the phase contrast micrograph and HCS Red stained image (Figure 1A, lower panel), and cell aggregates visible in PC12 were conspicuously absent in NS-1. Quantitative assessments of neurite lengths and branch points by progressively increasing the concentration of NGF confirmed a rapid dose-dependent response in neurite outgrowth, sustained neurite elongation, and increased neurite branch points in NS-1 at 48 h post NGF treatment. As little as 3.13 ng/mL of NGF was sufficient to induce quantifiable neurite outgrowth at 48 h in NS-1 cells, achieving a plateau at approximately 12.5 ng/mL NGF (Figure 1B). The maximum neurite length of ~8 μm recorded for NS-1 was 8-times greater than the length of neurites in PC12 and the branch points in NS-1 were 10-fold more than PC12. Although differentiation of PC12 resulted in neurite outgrowth the lengths did not change significantly with increasing concentrations of NGF up to 50 ng/mL, and very few branch points were detected (Figure 1C). To evaluate the proliferative capacity, the growth profile of NS-1 and PC12 were measured at various time points up to 72 h in culture. We observed no significant differences in cell proliferation up to 35 h as measured by cell viability assay (Figure 1D). However, clear divergence in cell proliferation was seen beginning at ~40 h in NS-1 relative to PC12, culminating in a 2-fold difference in fluorescence intensity at 72 h. The results of these experiments clearly demonstrate an increased sensitivity of NS-1 to NGF as observed by accelerated formation of neurites, neurite elongation, increased neurite networks, and enhanced proliferation compared to PC12.

### Transcriptional analysis of neuronal markers in naïve and NGF differentiated cells

**Figure 2:**
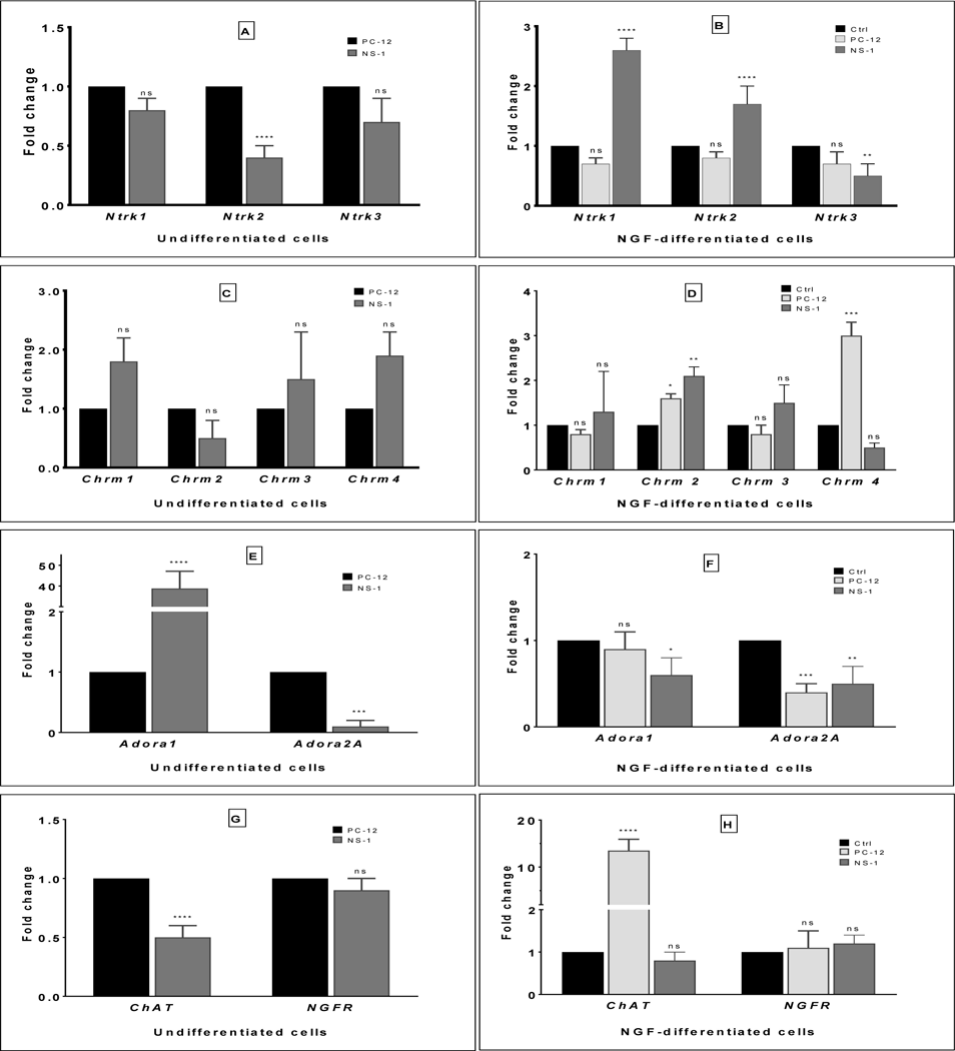
Analysis of transcription patterns of neuronal genes. Transcription of the neuronal marker genes Ntrk, Chrm, Adora, Chat, and Ngfr were measured in naïve and NGF exposed NS-1 and PC12 cells. Fold change in gene transcripts are shown as bar graphs in (A) undifferentiated and (B) NGF-differentiated Ntrk genes (Ntrk1, Ntrk2, and Ntrk3); (C) undifferentiated and (D) NGF-differentiated Chrm genes (Chrm1, Chrm2, Chrm3, Chrm4); (E) undifferentiated and (F) NGF-differentiated Adora genes (Adora1, Adora2A); (G) undifferentiated and (H) NGF-differentiated ChAT and Ngfr genes. The data shows the means ± SD of at least three biological replicates (*p<0.05; **p<0.01; ***p<0.001; ****p<0.0001; indicates relative significance. ns=not significant).

To ascertain the identity relationships of NS-1 and PC-12 cell lines, transcriptional analyses of gene profiles in undifferentiated and NGF differentiated cells were assessed by RT-qPCR. Genes that establish the identity of neuronal cells including tyrosine kinase (*Ntrk*) receptors, muscarinic receptors and cholinergic receptors were analyzed (Figure 2). The *Ntrk2* transcript was reduced ~2-fold in undifferentiated NS-1 compared to PC12 cells. In contrast, when cells were differentiated with NGF, the transcription of *Ntrk1* and *Ntrk2* were highly upregulated in NS-1 by ~2.5-fold and ~1.5-fold respectively, while *Ntrk3* was significantly decreased compared to untreated cells (Figure 2B). No change occurred in the transcription of *Ntrk* genes in differentiated or undifferentiated PC12. When expression of muscarinic receptors was quantified, we observed no statistically significant differences in any *Chrm* gene profile between the two cell lines in undifferentiated state (Figure 2C). After treatment with NGF, differentiation of the cells was accompanied by significant upregulation of *Chrm2* alone in both cells with NS-1 expressing ~2-fold (p<0.01) increase of *Chrm2* while PC12 showed a modest but significant increase in transcription of the *Chrm2* (Figure 2D). In contrast, the transcription of *Chrm4* revealed a highly dissimilar pattern of regulation between NGF-treated cells. *Chrm4* increased ~3-fold (p<0.001) compared to unexposed cells, whereas, no change in gene transcription was observed in NS-1. Interestingly, the increase of *Chrm4* detected in undifferentiated NS1 was significantly inhibited by NGF treatment relative to background expression of untreated PC12 cells.

Analysis of *Ntrk* genes in undifferentiated cells revealed no statistical difference (p>0.05) in *Ntrk1* and *Ntrk3* but showed a significant difference in transcriptional regulation of *Ntrk2* (p<0.001) (Figure 2A).

Analysis of adenosine gene transcripts showed high a degree of difference in the level of transcripts of two receptor subtypes analyzed in NS-1. A 40-fold (p<0.0001) increase in *Adora1* mRNA was detected in NS-1 untreated with NGF relative to PC12, while *Adora2A* transcript was barely detectable in the same cell line (Figure 2E). NGF-treatment was accompanied by a massive reduction in the magnitude of *Adora1* transcript in NS-1 to nearly one-half that of untreated control with no alteration in transcript level in PC12 (Figure 2F). While *Adora2A* transcript was undetectable in naïve NS-1, NGF exposure led to an increase in transcript of ~50% (p<0.01) of untreated control. Interestingly, differentiation of PC12 with NGF resulted in significant reduction in *Adora2* mRNA, although the transcript levels in both cell lines were significantly less than undifferentiated cells.

Finally, we assessed the transcript abundance of two cholinergic markers, *ChAT*, and the low affinity neurotrophin receptor NGFR/*p75*^*NTR*^. In control cells not exposed to NGF, the transcript level of *ChAT* was significantly less in NS-1 at ~50% (p<0.0001) the magnitude in PC12 (Figure 2G). However, NGF differentiation led to ~14-fold (p<0.0001) increase of *ChAT* gene activity in PC12 but had modest effect in NS-1 (Figure 2H). NGFR gene activity as neither altered in undifferentiated nor differentiated forms of NS-1 and PC12.

### Immunoblot analysis for expression of cholinergic markers in NS-1 and PC12 cell lines

**Figure 3:**
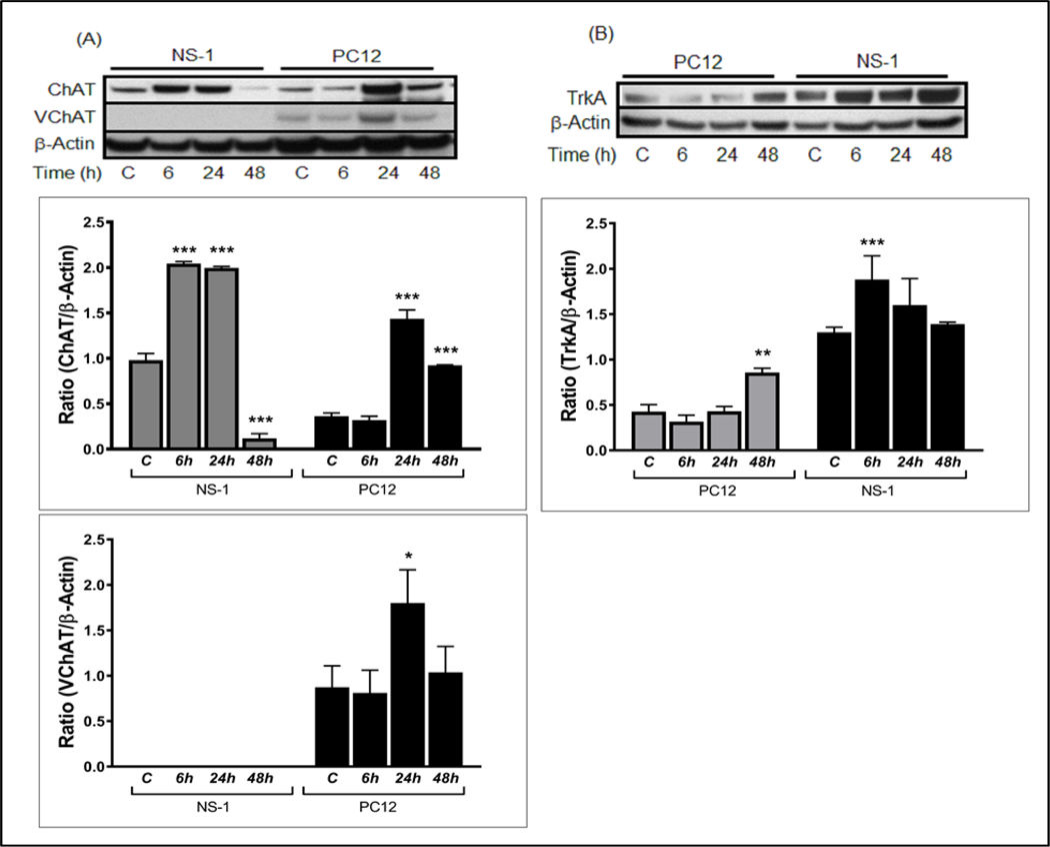
Evaluation of neuronal protein expression in differentiated NS-1 and PC12 cells by immunoblot analyses. Cells were treated with NGF for 6 h, 12 h, and 24 h and the cellular lysates subjected to western blotting with specific antibodies to (A) ChAT and VAChT, and (B) TrkA proteins. Quantification of the relative expression of ChAT, VAChT, and TrkA is provided in the bar graphs. β-actin was used as an internal control of protein expression. (*p<0.05 and ***p<0.001 indicates significance compared with untreated controls labeled C in the figure).

Our investigation of transcription of neuronal indicators showed significant changes in gene transcription patterns of naïve and NGF-differentiated cells in NS-1 and parental PC12 cell lines. To verify whether changes observed in gene transcription were translated to protein expression, we measured the amount of proteins in both naïve and NGF differentiated cells by immunoblot. The molecules analyzed included ChAT, VAChT, and TrkA (Figure 3).

We found that NS-1 and PC12 cells both showed detectable expression of ChAT in undifferentiated state. However, the basal expression of ChAT protein in NS-1 was markedly higher than the basal expression in PC12 (Figure 3A). NGF treatment of NS-1 led to 2-fold (p<0.001) elevation in ChAT expression within 6 h in culture and the amount of protein detected in the cell remained unaltered at 24 h post NGF treatment, then subsequently decreased 9-fold (p<0.001) at 48 h compared control cells. Likewise, differentiated PC12 showed significant 4-fold (p<0.001) increase of ChAT expression relative to untreated PC12 but at protracted time point (24 h) relative to upregulation in NS-1 which occurred in 6h. Unlike NS-1 which showed nearly undetectable ChAT protein at 48 h, the level of ChAT in NGF-differentiated PC12 at 48 h decreased to a certain extent but was very robust at 2.6-fold (p<0.001) relative to the control PC12.

Meanwhile analysis of the degree of expression of VAChT showed that the protein was not expressed in undifferentiated or NGF differentiated NS-1 cell (Figure 3A). In contrast, VAChT protein was detected in untreated PC12, although in minute quantity. Similarly, the amount of VAChT detected in PC12 cell subjected to NGF treatment for 6 h was not significantly different to baseline expression seen in control cells. However, at 24 h post NGF differentiation, the expression of VAChT was significantly elevated by 2.1-fold (p<0.05) compared to undifferentiated group. The pronounced increase at 24 h was followed by a reduction to near basal expression with continued incubation for 48 h. We also investigated the influence of NGF on the amount of Trk receptor protein in the cells. Figure 3B demonstrates that cells which were not exposed to NGF had considerable expression of TrkA, but the expression was more pronounced in NS-1 which exhibited a 2-fold increase in TrkA content relative to PC12. Treatment with NGF significantly increased the concentration of TrkA in NS-1 at 6 h, remaining relatively constant or slightly reduced at 24 h and 48 h. PC12 cells on the other hand showed no change expression of TrkA under NGF differentiation at 6 h and 24 h compared to control. However, a 2-fold (p<0.05) increase in the protein concentration of TrkA was detected in PC12 at 48 h post NGF exposure, but the increase was less than the basal expression in undifferentiated NS-1 cells.

## Discussion

The use of *in vitro* cellular models has played a significant role in understanding the molecular and cellular processes relevant to disease pathogenesis and disease progression. PC12, a pheochromocytoma cell of adrenal medulla has proved a useful model system to study the mechanisms of neuronal growth, proliferation, differentiation, communication, injury and death for several decades. For several decades PC12 has proved a useful model system for studying neurodegenerative diseases including AD and Parkinson’s [33,34]. However, this model has been confounding with several limitations that include time-consuming culture methods, protracted doubling time and long differentiation period. NS-1, a derivative cell line of PC12 has been gaining traction as an alternative model for phenotypic and molecular investigations that evaluate chemical effects on neurite outgrowth due to its enhanced sensitivity to NGF, accelerated doubling time, ease of handling, and inherent property to form elaborate neuritic processes in culture with NGF differentiation [2,5,30,35]. Differences in gene expression profile amongst diverse subclones of SH-SY5Y neuroblastoma cells have been determined to play significant roles in modulating neurite outgrowth responses to NGF [36]. Given that both NS-1 and the parent cell line PC12 exhibit similar morphometric and phenotypic properties but on a much different time scale, we sort to investigate the differences in molecular architecture of the cells that could potentially account for these differences.

Trk receptors belong to a large family of membrane-spanning proteins that possess intrinsic kinase activity. Trks are essential for development and maintenance of the nervous system and control a large cascade of signaling processes that regulate cell survival, differentiation, proliferation, axonal elongation, neurite outgrowth, and synaptogenesis upon activation by growth factors [37–39]. The activation of Trk receptors by neurotrophins have been implicated in promoting survival and inducing apoptosis in PC12 [40,41]. Consequently, we assessed whether NS-1 cells express *Trk* receptors and compared the levels of expression to PC12 by gene expression analysis. Our results demonstrate that NS-1 expresses all three *Trk* receptor genes in undifferentiated state. Expression of *Ntrk* is crucial for the normal development and function of the nervous system. TrkA, the principal receptor for NGF plays important physiological role in the function of sympathetic neurons and is necessary for their survival and differentiation in vitro and in vivo [42]. We observed highly elevated transcription of *Ntrk1* and *Ntrk2* in NS-1 cells following NGF differentiation, whereas the transcription in PC12 was unaltered. NGF is well-known to exert its neurotrophic actions via intracellular signaling mechanisms that lead to survival, proliferation and differentiation. The binding of TrkA receptor by NGF activates downstream signaling responses controlled by Ras/MAPK, phosphatidylinositol 3-kinase (PI3K)/Akt, and phospholipase C (PLC)-γ. Akt phosphorylation activates downstream genes to regulate neuronal survival either by inducing the transcription of survival genes or by inhibiting apoptosis. Overexpression study in PC-12 and SH-SY5Y suggest that NGF-induced increases in TrkA mRNAs expression is directly related to the formation of neurites [36,43]. *Ntrk2* which encodes TrkB is upregulated in presence of BDNF and phosphorylates neural cell adhesion molecule (NCAM). However, TrkB can also interact with NCAM in absence of BDNF [44] and induces neurite outgrowth in hippocampal neurons. Given that NGF is the primary ligand of TrkA, our data imply that the marked change in *Ntrk1* gene expression in addition to *Ntrk2* in NGF-differentiated NS-1 suggests a role, to a certain extent, for the enhanced sensitization and rapid activation of differentiation program in NS-1 immediately after NGF treatment.

Muscarinic receptor signaling have been proposed to coordinate the response of neural networks in the CNS and thus, play an important role during brain development by regulating cell survival, proliferation, and differentiation. The stimulation of neuronal muscarinic receptors has been shown to induce neurite outgrowth in a variety of neuronal cell lines [45,46] NGF differentiation of PC12 has also been previously reported to be accompanied by a large increase in the expression of muscarinic receptors [47]. Therefore, we investigated whether differences existed in the transcriptional regulation of *Chrm* gene family that could potentially indicate a role of these genes in phenotypic variations between NGF differentiated NS-1 and PC12 cells. NGF differentiation increased the transcription of *Chrm2* in both cell lines. However, the regulation of *Chrm4* was inversely correlated in the cell lines relative to cells unexposed to NGF, with *Chrm4* increasing greater than 3-fold in PC12. NGF has been previously been reported to upregulate *Chrm2* receptor in PC12 potentially through inward rectifying potassium channel Kir3.2. In addition, NGF differentiated PC12 was shown to regulate m4 receptor mRNA stability through MAP kinase activity. Our findings are in agreement with these earlier reports on regulation of *Chrm* genes in PC12. The inverse correlation of *Chrm* gene transcripts in NS-1 point to complexities in the mechanisms that control transcription of *Chrm* genes in this cell line and could potentially be involved in surmounting the temporal constraints of NGF differentiation of NS-1 cells relative to the parental PC12. However, the role of these genes in the rapid changes in NS-1 subject to NGF treatment remains to be systematically investigated.

The adenosine receptor has been proposed to control NGF-induced neurite outgrowth in PC12 and primary hippocampal neuron [28]. Adora1 and Adora2A receptors which are present in neurons demonstrate opposing actions in the CNS and play significant roles in the regulation of neurotransmitters. In our study, we found that NS-1 cells differentially transcribed *Adora1* and *Adora2A* receptor mRNA in native state. These differences were normalized by NGF treatment. We also found that NGF-induced differentiation of NS-1 was accompanied by a highly significant down-regulation of Adora1 gene transcription. The decrease in *Adora1* and *Adora2A* regulation observed in our study after treatment with NGF is consistent with previous research in PC-12 [48–50]. Adora1 receptor activation was shown to inhibit Rho-mediated formation of neurite outgrowth in PC12 [51]. Therefore, it is likely that the vast reduction in activity of *Adora1* gene (~80-fold) following NGF differentiation could have implication in the compressed timeline leading to rapid neuritogenesis, development of neurites, and formation of neuritic branch points by NS-1.

Acetylcholine (Ach), a neurotransmitter which promotes neuronal differentiation, is synthesized from choline and acetyl coenzyme A in a one-step reaction catalyzed by ChAT. Increase in ChAT mRNA is normally associated with differentiation of PC12 by NGF. Also, transfection of ChAT cDNA in the neuroblastoma cell N18TG2 lead to increased capacity of the cell to grow fibers, express synapsin 1, and induced neurites with carbachol treatment [46]. In our experiments we observed a 12-fold increase *ChAT* mRNA inPC12 that was not seen in NS-1 after treatment with NGF. It is uncertain why the level of *ChAT* transcript was unaffected in NS-1 given prior observations in parental PC12. However, muted response in mRNA content of NS-1 could suggest a possible role for ChAT that is limited to cellular events without prime contribution to morphological changes associated with neurite outgrowth and elongation. This is unlikely because the protein content of ChAT was markedly upregulated in NS-1 after NGF exposure which indicates that expression of ChAT is controlled post-transcriptionally. Our data clearly suggests the operation of altered mechanisms for the control of ChAT expression in NS-1 and PC12 and warrants further enquiry.

PC12 cells synthesize and store catecholamines including dopamine and norepinephrine. They exhibit sympathetic neuronal properties and increase the expression of cholinergic markers, ChAT, and Ach activity in the presence of NGF [52–53]. NGF induces cholinergic differentiation by strongly elevating the expression of ChAT and increasing intracellular ACh levels through the PI3-kinase pathway [16]. In our study, we saw the first evidence of significant increase in ChAT protein expression in NS-1 at 6 h post NGF exposure. The increase was sustained for 24 h and was barely detectable at 48 h. In contrast, a significant increase in ChAT protein was detected in PC12 at 24 h after NGF treatment. Unlike NS-1, ChAT protein expression was not sustained in PC12 but declined considerably at 48 h, notwithstanding a 14-fold increase in transcriptional activity of *ChAT* in PC12. Although we did not evaluate the protein levels of Ach in the cells, the early induction of ChAT expression in NS-1 following NGF exposure would predict a cognate increase in ACh as reported in the literature for PC12 and thus, drive early differentiation events in this cell model relative to PC12. ChAT is the main ACh synthesizing enzyme and its activity is strongly correlated and in balance to ACh levels. Enhanced secretion of ACh is essential for neurotransmission and may play a role in the activation of transcription factors such as early growth response protein 1 (EGR-1) essential for neurogenesis [54]. The existence of cross-talk has been established between several neurogenic pathways and studies have revealed association between TrkA and other cholinergic markers. Increased *ChAT* and *VAChT* mRNA have been observed in cells transfected with TrkA, while TrkA and ChAT proteins showed similar distribution pattern on the forebrain neurons during postnatal period. We observed peculiar differences in the expression of ChAT and VAChT in NS-1 andPC12. VAChT was expressed in PC12 and its regulation paralleled the expression of ChAT. In contrast, no evidence of VAChT expression was seen in differentiated NS-1 cells measured by immunoblot. The absence of VAChT in NS-1 is intriguing because it suggests the likelihood that distinct pathways exist for synthesis and secretion of ChAT in this cell line, similar to NG108–15 cells [55]. The inverse regulation between ChAT and VAChT at the gene and protein levels in NS-1 is highly is thought-provoking and merits further studies as they could be potentially key determinants in the robust morphological and phenotypic characteristic exhibited by the cell line. Taken together our results imply that robust differences in NGF-dependent transcriptional controls of *Trk*, *Chrm*, *Adora*, and *ChAT* genes and associated translational regulation of Trk, ChAT, and VAChT protein expression potentially affect the responsive growth profiles, morphological attributes, and phenotypic properties of NS-1 and establishes NS-1 as a valid surrogate cell model for cholinergic neuronal research.

## Conclusion

Drug discovery for modulators of neuronal phenotypes uses *in vitro* testing of compound libraries and complex imaging systems as primary screening tools. Therefore, the choice of cell line is critical when neurite outgrowth is the output criteria for analysis.PC12 cell line, the widely used model for neuronal differentiation studies presents numerous challenges in discovery and research environments due to prolonged culture and differentiation duration, growth requirements, and the tendency to form aggregates. Here we showed that NS-1 cell, aderivative of PC12 exhibits enhanced characteristics relative to differentiation, growth and phenotypic properties in a shorter timeframe compared to the parental cell line. In addition, NS-1 cells express neuronal markers following differentiation with NGF. These characteristics support the emerging use of NS-1 as an alternate in vitro neuronal cell model.

## Figures and Tables

**Table 1.** Sequences of primers used for PCR

Name	Forward Sequence (5’→3’)	Reverse Sequence (5’→3’)	Length
*Ntrk1*	AGCACAGACTACTACCGTGTGG	CACATCACTCTCGGTGCTGAAC	22
*Ntrk2*	TTCCAAGTTTGGCATGAAAGGC	CGAAGAAGACGGAGTGTTGCTC	22
*Ntrk3*	TGCCTGATGTGGACTGGATA	TGTCTTCGCTCGTCACATTC	20
*Chrm1*	AACATCACTGTCTTGGCACCAG	ACCAGTAGGTTGCCTGTCACTG	22
*Chrm2*	ATACCCTCTACACTGTGATTGGC	TTCATAACGGAGGCATTGCTGAC	23
*Chrm3*	ATGCCTCTGTCATGAATCTGCTG	GCCAGACCAATCATCACACCAC	23
*Chrm4*	CCAGAGCACAAAGGACAAGACC	CCAGAGCACAAAGGACAAGACC	22
*ChAT*	CTCCCCAAAAGATGCCTGTA	CTGGCTCTTCCTGAACTGCT	20
*NGFR*	CACCGACAACCTCATTCCTGTC	TTGTTTGCAGCTGTTCCACCTC	22
*Adora1*	AGATCCCTCTCCGGTACAAGAC	AGCCAAACATGGGTGTCAGG	22
*Adora2A*	AGCCAGAGCAAGAGGTATCTCAG	CGAAGACGTTCTCACAGACGAC	23
